# Understanding the Action of RARγ Agonists on Human Osteochondroma Explants

**DOI:** 10.3390/ijms21082686

**Published:** 2020-04-13

**Authors:** Sonia A. Garcia, Hongying Tian, Yuka Imamura-Kawasawa, Aidan Fisher, Ashley Cellini, Casey Codd, John E. Herzenberg, Joshua M. Abzug, Vincent Ng, Masahiro Iwamoto, Motomi Enomoto-Iwamoto

**Affiliations:** 1 Department of Orthopaedics, University of Maryland School of Medicine, 20 Penn Street, HSFII, Baltimore, MD 21201, USA; Sonia.garcia@som.umaryland.edu (S.A.G.); htian@som.umaryland.edu (H.T.); afishe1@umbc.edu (A.F.); ACellini@som.umaryland.edu (A.C.); CCodd@som.umaryland.edu (C.C.); Jherzenb@lifebridgehealth.org (J.E.H.); jabzug@som.umaryland.edu (J.M.A.); vng@som.umaryland.edu (V.N.); Masahiro.Iwamoto@som.umaryland.edu (M.I.); 2 Departments of Pharmacology and Biochemistry and Molecular Biology, Institute for Personalized Medicine, Pennsylvania State University College of Medicine, Hershey, PA 17033, USA; yimamura@pennstatehealth.psu.edu; 3 Pediatric Orthopaedics, Sinai Hospital, Baltimore, MD 21215, USA

**Keywords:** osteochondroma, retinoic acid nuclear receptor, growth plate, transcriptome analysis

## Abstract

Osteochondromas are cartilage-capped growths located proximate to the physis that can cause skeletal deformities, pain, limited motion, and neurovascular impingement. Previous studies have demonstrated retinoic acid receptor gamma (RARγ) agonists to inhibit ectopic endochondral ossification, therefore we hypothesize that RARγ agonists can target on established osteochondromas. The purpose of this study was to examine the action of RARγ agonist in human osteochondromas. Osteochondroma specimens were obtained during surgery, subjected to explant culture and were treated with RARγ agonists or vehicles. Gene expression analysis confirmed the up-regulation of RARγ target genes in the explants treated with NRX 204647 and Palovarotene and revealed strong inhibition of cartilage matrix and increased extracellular matrix proteases gene expression. In addition, immunohistochemical staining for the neoepitope of protease-cleaved aggrecan indicated that RARγ agonist treatment stimulated cartilage matrix degradation. Interestingly, cell survival studies demonstrated that RARγ agonist treatment stimulated cell death. Moreover, RNA sequencing analysis indicates changes in multiple molecular pathways due to RARγ agonists treatment, showing similarly to human growth plate chondrocytes. Together, these findings suggest that RARγ agonist may exert anti-tumor function on osteochondromas by inhibiting matrix synthesis, promoting cartilage matrix degradation and stimulating cell death.

## 1. Introduction

Osteochondroma is the most common benign bone tumor in children. Osteochondromas are cartilage-capped tumors that are located proximate to the growth plate and can be either solitary or multiple osteochondromas (MO) [1,2]. Developing osteochondromas can cause skeletal deformities and disturbance on neurovascular structures or nearby tendons causing discomfort and limited motion [3,4,5]. Osteochondromas are unresponsive to chemotherapy and radiation, making surgery the only available treatment [3,4,5,6]. The anatomical location of osteochondroma prolongs surgery since it requires the mobilization of neurovascular and other critical structures away from the existing osteochondroma [5,7,8]. There are many risks associated with surgery and the development of a pharmacological therapy is a promising non-surgical alternative to potentially prevent the development and/or treat existing osteochondromas [5]. 

MO is associated with a hereditary syndrome known as Multiple Hereditary Exostosis (MHE) [3,6]. The majority of MHE patients have autosomal-dominant functional mutations in *Exostosin1* (*EXT1*) and/or *Exostosin2* (*EXT2*) genes [3,6]. The *EXT1* and *EXT2* genes encode endoplasmic reticulum-localized type II transmembrane glycoproteins that are tightly associated with glycosyltransferase activities, critical for heparan sulfate proteoglycan biosynthesis [9,10,11]. The pathogenesis of osteochondroma development is not fully elucidated, however, heparan sulfate synthesis deficiency likely underlies the molecular mechanism of osteochondroma formation. Since osteochondromas are located proximate to the growth plate, osteochondromas may potentially arise during skeletal development from the growth plate itself or neighboring connective mesenchymal cells due to the dysregulation of chondrogenesis and/or endochondral ossification. 

Retinoic acid receptor gamma (RARγ) is an important regulator of cartilage development and growth [12,13]. Stimulation of RARγ action by retinoid agonists has been shown to inhibit heterotopic ossification in various types of animal models [14,15,16]. These preclinical studies led to a clinical trial investigating Palovarotene, a RARγ agonist as a potential treatment for Fibrodysplasia ossificans progressiva (FOP), which is characterized by heterotopic bone formation leading to progressive loss of mobility and function. In addition, preclinical studies have also demonstrated that stimulation of RARγ suppresses chondrogenesis and osteochondroma formation in MHE mouse models via BMP signaling inhibition [17]. A clinical trial is currently underway investigating the efficacy and safety of Palovarotene for MO in 240 pediatric subjects (https://clinicaltrials.gov/ct2/show/NCT03442985). In order to offer efficient and safe therapies in the upcoming future for MO, understanding the molecular actions of RARγ agonists on human osteochondromas is vital. 

The purpose of this study was to evaluate the pharmacological effects of the RARγ agonists on human osteochondromas. Since RARγ agonist strongly affects the function of mouse growth plate chondrocytes [12] and inhibits the formation of osteochondromas in mice [17], we hypothesized that RARγ agonist can act on established human osteochondromas. Human osteochondromas were obtained during surgery and subjected to RARγ agonists treatment via explant culture followed by histological and transcriptome analysis. 

## 2. Results

### 2.1. Osteochondroma Explant Cultures

**Figure 1:**
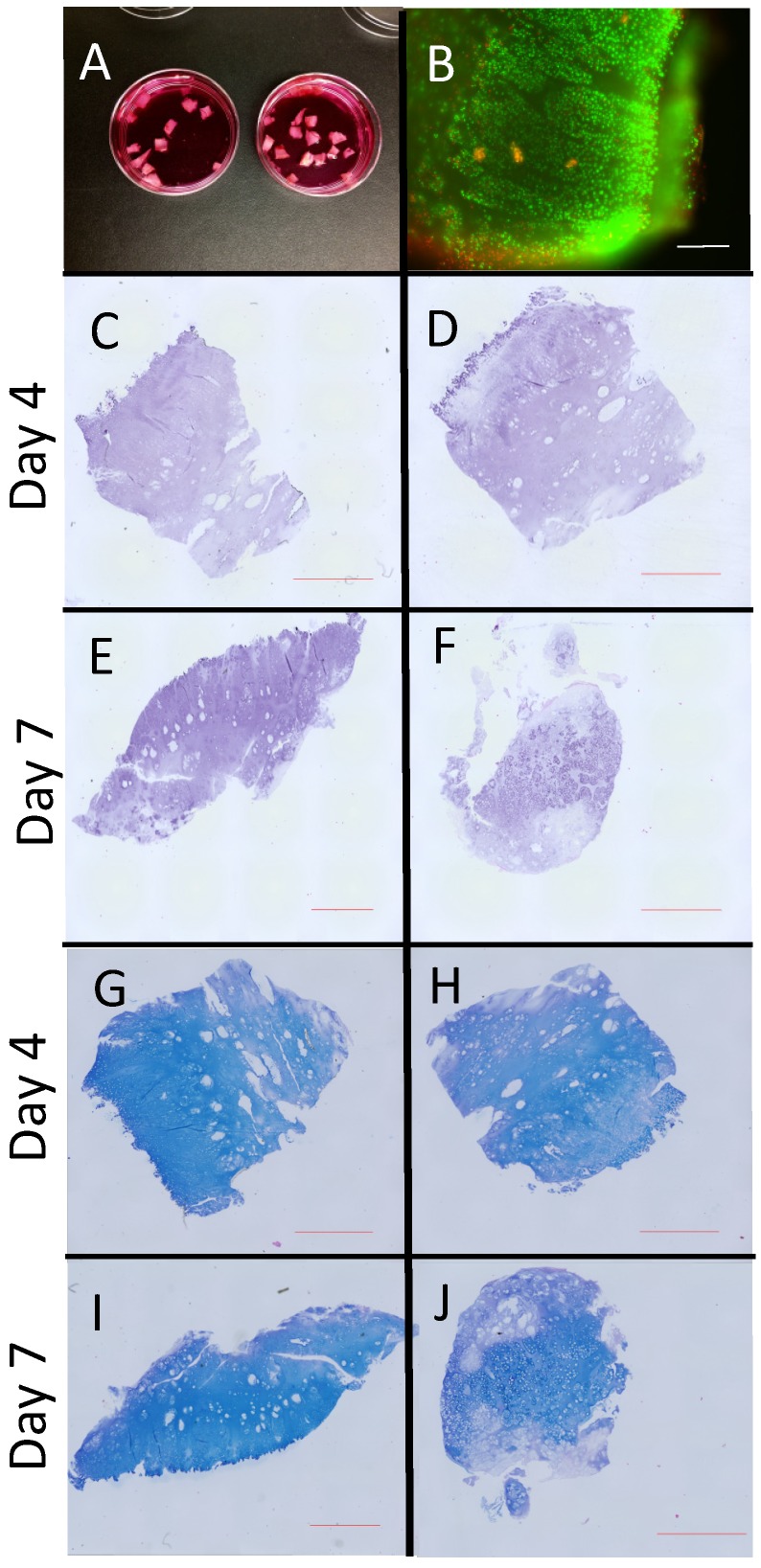
Osteochondroma explant cultures. Cartilaginous portions (cartilage caps) were micro-dissected from human osteochondromas excised at surgery and made into 3–5 mm cubic (**A**) and subjected to explant culture. On the next day, the explants were subjected to Live/dead assay (**B**) or treated with vehicle (0.1% ethanol) (**C**,**E**,**G**,**I**) and 300 nM NRX204647 **(D**,**F**,**H**,**J)** in in Dulbecco’s Modified Eagle Medium (DMEM) containing 2% charcoal-treated FBS. The explants were fixed with 4% PFA 4 day (**C**,**D**,**G**,**H**) or 7 day (**E**,**F**,**I**,**J**) after treatment, and then stained with hematoxylin-eosin (**C**–**F**) or alcian blue (**G**–**J**)**.** Bars are 600 μm for **B** and 2 mm for **C**–**J**.

Cartilage caps were excised from osteochondroma specimens (Table S1), dissected to create 3–5 mm^3^ pieces (Figure 1A) and subjected to explant culture. Cell viability was evaluated by live/dead assay on Day 1, confirming that most of the cells were alive except for cells at the periphery (Figure 1B). Osteochondroma explants were treated with RARγ agonists (NRX204647 or Palovarotene) for 4 or 7 days. Hematoxylin-eosin staining of osteochondroma explants did not show significant changes in overall view seven days after NRX204647 RARγ agonist treatment but did however show an evident decrease in the staining intensity in RARγ-agonist treated groups (Figure 1C–F). Reduction in proteoglycan matrix was also visualized by alcian blue staining in the RARγ treated explants on Day 7 compared to control (Figure 1G–J).

### 2.2. Comparison of Gene Expression Profile between Control and RARγ Agonist Treated Osteochondromas

**Figure 2:**
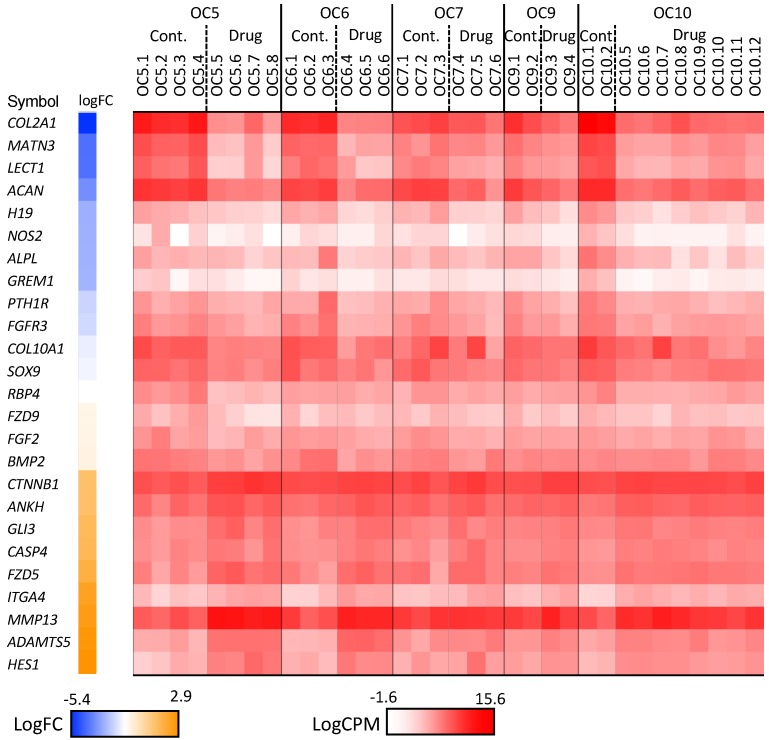
Log counts per million (CPM) histogram of genes listed in the osteoarthritis pathway identified by the IPA. The RNAseq data from OC5, OC6, OC7, OC9 and OC10 were combined and analyzed. A total of 395 genes were up-regulated and 598 genes were down-regulated. The DEGs (criterion false discovery rate (FDR) ≤ 0.05 and the log2 fold change (LFC) cutoff values of +/− 1) between retinoic acid receptor gamma (RARγ) agonists treatment and control groups were analyzed to identify canonical pathways by IPA. Heatmap indicates the sample differences of DEGs listed in the osteoarthritis pathway. OC5: OC5.1–5.4, control; OC5.5–5.8, 300 nM NRX204647. OC6: OC6.1–6.3, control; OC6.4–6.6, 300 nM NRX204647. OC7: OC7.1–7.3, control; OC7.4–7.7, 300 nM NRX204647. OC9: OC9.1 and 9.2, control; OC9.3 and 9.4, 300 nM NRX204647. OC10: OC10.1 and 10.2, control; OC10.5 and 10.6, 50 nM NRX204647; OC10.7–10.9, 300 nM NRX204647; OC10.10–10.12, 300nM Palovarotene.

To understand how RARγ agonist treatment affects biological functions in osteochondroma cells, we performed transcriptome analysis. Five independent patient specimens were subjected to RNA sequencing analysis, and differential gene expression between control and RARγ agonist treatment were compared. We used DAVID [18,19] to identify GO-enriched functions for DEGs (differentially expressed genes). A total of 395 genes were significantly up-regulated and 598 genes were down-regulated between drug and control treatment groups. These DEGs satisfied both false discovery rate (FDR) < 0.05 and log2 fold change (LFC) with cutoff values of +/− 1 (Table S2). Collectively, these DEGs showed significant enrichments in extracellular matrix organization, skeletal system development, collagen fibril organization, cell adhesion, and collagen catabolic process (Table 1, Table S3). The Ingenuity Pathway Analysis (IPA) demonstrated that these DEGs were highly linked to hepatic fibrosis, GP6 signaling, atherosclerosis signaling, osteoarthritis, and interferon signaling pathways (Table 2, Table S4). A comparative analysis of each individual sample between the control and drug-treated groups showed DEG enrichment in similar canonical pathways, including GP6 signaling, hepatic fibrosis, and osteoarthritis pathways (Table S5). We particularly studied the osteoarthritis pathway because the subsequent target genes are involved with cartilage structure and function. The changes in expression of the DEGs listed in the osteoarthritis pathway included strong down-regulation of cartilage matrix molecules (*COL2A1*, *MATN3*, and *ACAN*) and up-regulation of matrix proteases (*ADAMTS5* and *MMP13*) and apoptosis-related molecules (*CASP4*) (Figure 2). 

**Figure 3:**
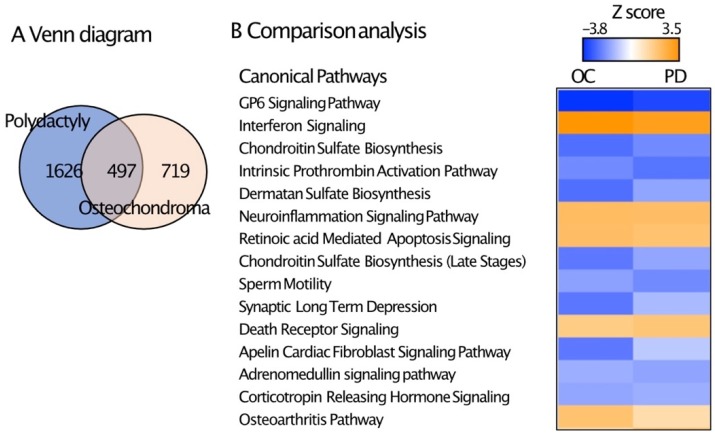
Comparison of DEGs between control and RARγ-agonist treatment groups of osteochondroma explants with those of polydactyly chondrocyte cultures. The DEGs (criterion FDR ≤ 0.05 and the LFC cutoff values of +/− 1) between RARγ agonists treatment and control groups were analyzed in the osteochondroma explant cultures (OC10) and human polydactyly chondrocytes. (**A**) Venn diagram analysis of DEGs of osteochondroma explants and polydactyly chondrocyte cultures. (**B**) Canonical pathways highly ranked by the comparative analysis of IPA.

To compare the effect of RARγ agonists between osteochondromas and human growth plate chondrocytes, we obtained chondrocytes from polydactyly samples, since the acquisition of enough amounts of chondrocytes from normal growth plate is limited. Interestingly, 698 genes were significantly up-regulated and 1425 genes were down-regulated between NRX204647 and control treatment groups (Table S6). The canonical pathway analysis by IPA revealed that DEGs were also highly linked to the osteoarthritis pathway (Table 3, Table S7). We performed a comparative analysis of DEGs between osteochondroma cells and polydactyly chondrocytes and were subjected to RNA sequencing parallelly. Results were categorized in Venn diagrams (Figure 3A). 497 genes were found to be in common between the 2 groups. In addition, the common pathways highly ranked by IPA comparison analysis include GP6 signaling, interferon signaling and several types of proteoglycan biosynthesis pathways (Figure 3B).

**Figure 4:**
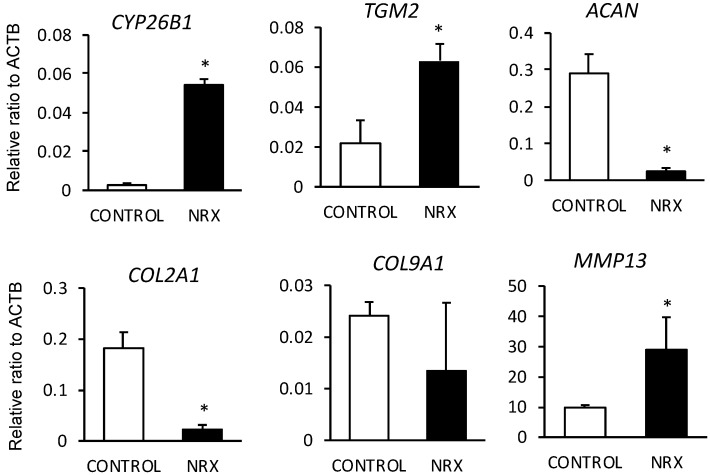
Gene expression analyses of RARγ agonist treatment and control groups of osteochondroma explant cultures. The osteochondroma explants (hOC7) were treated with vehicle (CONTROL, 0.1% ethanol) and 300 nM NRX204647 (NRX) in DMEM containing 2% charcoal-treated FBS (*n* = 3). Total RNAs were prepared and subjected to qPCR to examine gene expression levels of *CYP26B1, TGM2*, *ACAN*, *COL2A1*, *COL9A1* and *MMP13*. The values were normalized by *ACTB* value and then calculated as a relative ratio to the control average. The values are average and standard deviation (SD). * *p* < 0.05 versus control.

RNA sequencing results were then validated via qPCR analysis for cartilage matrix molecules and cartilage matrix proteases using independent patient samples. RARγ target genes, *TGM2* and *CYP26B* were up-regulated in osteochondromas (Figure 4 and Figure S1). In addition, gene expression levels of *ACAN, COL2A1, COL9A1* and *SOX 9* were strongly reduced while levels of matrix proteases, such as *MMP13* were up-regulated. 

### 2.3. RARγ Agonists Induce the Actions for the Destruction of Osteochondroma Cartilage

**Figure 5:**
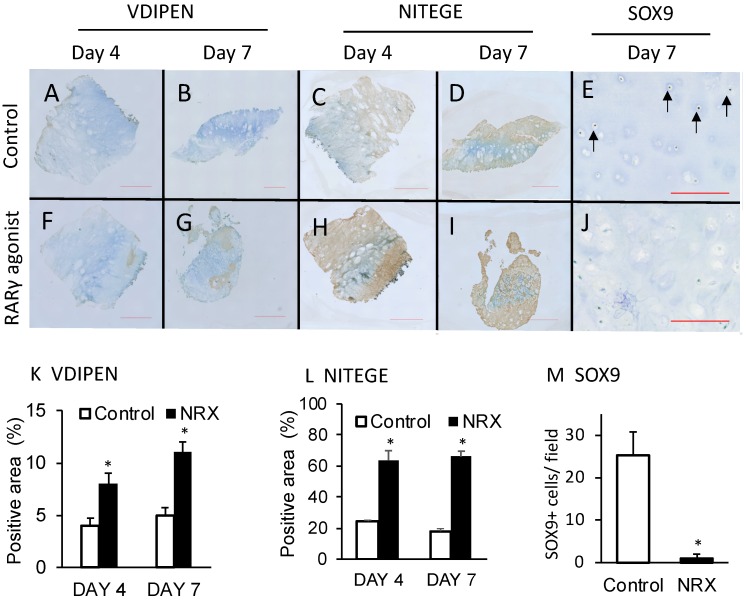
Stimulation of aggrecan degradation and inhibition of SOX9 expression by RARγ agonists in osteochondroma explant cultures. The osteochondroma explants were treated with vehicle (Control, 0.1% ethanol) (**A**–**E**) or 300 nM NRX204647 (RARγ agonists) (**F**–**J**) in DMEM containing 2% charcoal-treated FBS (*n* = 3). The explants were fixed with 4% PFA 4 or 7 days after treatment and subjected to immunohistochemical staining using anti-*VDIPEN*, *NITEGE* or *SOX9* antibodies. The staining results were quantified by Cell Hybrid Count function in BZX-700 (**K**–**M**) The values are average and standard deviation (SD). * *p* < 0.05 versus control. Bars are 2 mm for A-I and 200 μm for (**E**) and (**J**).

**Figure 6:**
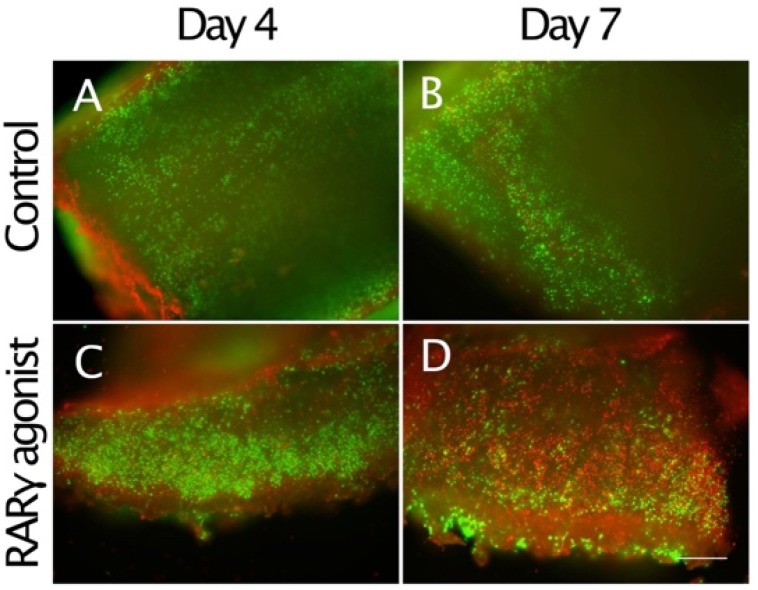
Stimulation of cell death by RARγ agonists in osteochondroma explant cultures. The osteochondroma explants were treated with vehicle (0.1% ethanol) (**A** and **B**) or 300 nM NRX204647 (**C** and **D**) in DMEM containing 2% charcoal-treated FBS (*n* = 2, 3 patients). The explants were subjected to Live/death assay 4 or 7 days after treatment. Note that a much larger number of cells incorporated red-fluorescent ethidium homodimer-1 in the RARγ agonist-treated culture on day 7. Bars are 600 μm for (**A**–**D**).

Alteration of the osteoarthritis pathway due to RARγ agonist treatment was associated with the up-regulation of catabolic genes (*ADAMTS5*, *HES1*, and *MMP13*), inhibition of cartilage matrix anabolic genes (*COL2A1*, *ACAN*, and *SOX9*) and the up-regulation of death genes (*CASP4*) (Figure 2). These results indicate that RARγ agonists tend to destroy the cartilage of osteochondroma explants. To further examine this finding, we stained the sections with antibodies (VDIPEN and *NITEGE*) against the neo-epitope of aggrecan produced by *MMPs* and *ADAMTSs*, respectively. An increase in staining for VDIPEN and NITEGE neoepitope of aggrecan was observed (Figure 5). In contrast, SOX 9 immunoreactivity was detected in the control sample while it was hardly visible in the RARγ-treated samples (Figure 5J). Interestingly, RARγ agonist treatment-induced cell death as demonstrated by a live/dead assay. An increase in the number of dead cells by the drug treatment was not detected on day 4 while it became very evident on day 7 (Figure 6).

## 3. Discussion

Preclinical studies have mostly explored the inhibition of RARγ agonists on chondrogenesis, the differentiation of mesenchymal cells into chondrocytes, which produce and maintain the extracellular matrix of cartilage. RARγ agonist administration regimen starts from the initial stage, just after induction of heterotopic ossification (HO) in the HO animal models [14,15,16]. In the osteochondroma mouse models, Palovarotene treatment initiated at the stage of osteochondromas formation [17]. Furthermore, mechanistic studies of RARγ agonists on inhibition of HO and osteochondromas also focused on chondrogenesis in rodent cells. There is an ongoing clinical trial examining Palovarotene as a potential treatment for multiple osteochondromas, however, understanding the precise molecular action of Palovarotene cannot be monitored in these patients. In contrast to previous studies, our current study targets the chondrocytes of established human osteochondromas. Osteochondroma specimens used in this study were obtained from pediatric patients that have a certain size of osteochondromas and needed surgical excision to cure or remit their clinical symptoms. The excised cartilage caps obtained from the osteochondroma specimens consisted of chondrocytes surrounded by a large amount of proteoglycan. These cartilage caps likely contained mesenchymal cells and progenitors for osteochondroma cells, however, we expect low quantities. Furthermore, we observed a similar response of the cultured human polydactyly chondrocytes. Therefore, we can assume that this study shows the sensitivity of osteochondroma chondrocytes to RARγ agonists.

As mentioned above, the excised cartilage caps consist of chondrocytes surrounded by a large amount of proteoglycan. This extracellular matrix of cartilage can possibly provide the template for the developing osteochondroma, allowing its growth. Here we determined the important role of RARγ agonist treatment on the reduction of proteoglycan in the osteochondroma explant culture as characterized by alcian blue staining, and strongly stimulated proteoglycan degradation as determined by the immunostaining of the neoepitope of aggrecan core proteins. The immuno-reactivity to the anti-NITEGE antibody was more evident than that to the anti-VDIPEN, further supporting that ADAMTS4/5 dependent degradation is more dominant to MMP-dependent degradation in the RARγ-treated explant culture. In addition, our results clearly demonstrated that RARγ agonist treatment-induced cell death. The explants gradually increased cell death in the periphery even in the control culture, but the drug-treated culture showed a strong increase in the number of dead cells. These biological responses induced by RARγ agonists treatment suggest the possibility that RARγ agonists affect mature osteochondromas, by inducing both destruction of their extracellular matrix structure and stimulating cell death. 

The biological actions detected in the explant cultures were further verified by transcriptome analysis. In accordance with the previous results, the expression of matrix molecules rich in cartilage was strongly suppressed. Down-regulated extracellular matrix molecules encompassed a wide range of molecules including collagens, aggrecan core protein, matrilin, tenascin, fibronectin and laminin. Furthermore, the resulting DEGs contained several extracellular matrix receptors and enzymes that are involved in proteoglycan biosynthesis. These findings indicate that RARγ agonist strongly affects extracellular matrix metabolism and that RARγ agonists behaves as an inhibitor of the anabolic process of cartilage matrix in osteochondroma cells. In addition, expression of matrix degradation enzymes, such as *ADMTS5, ADAMTS9* and *MMP13* was up-regulated, indicating that degradation of aggrecan and collagens is enhanced in the drug-treated explants, complementing the results of the immunostaining of the neoepitope of aggrecan core proteins. Similar suppression of anabolic actions and stimulation of catabolic actions on matrix metabolism were also detected in polydactyly chondrocytes treated with RARγ agonists. These actions of retinoid signaling have been reported in rodent and rabbit growth plate chondrocytes [12,20,21,22]. We verified similar effects on human growth plate chondrocytes, although chondrocytes in polydactyly may not show the exact same nature as normal growth plate chondrocytes. 

In this study, we focused on the alteration of the osteoarthritis pathway among other IPA canonical pathways. This pathway includes various genes responsible for cartilage structure and function. The DEGs in this OA pathway can be summarized by strong down-regulation of cartilage matrix molecules and up-regulation of matrix proteases and apoptosis-related molecules. The RARγ-agonist treatment significantly altered the osteoarthritis pathway, however, the IPA analysis did not give a significant level of positive or negative Z-score to determine up- or down-regulation of this pathway. Further studies need to be conducted to link RARγ-agonist treatment to a pro- or anti-osteoarthritis pathway action. 

The canonical pathway analysis found alterations of the retinoic acid-mediated apoptosis signaling. In this pathway, *IRF-1* was up-regulated by RARγ agonist treatment in both osteochondroma explant cultures and polydactyly chondrocytes. *IRF-1* is a transcription factor that can be induced by interferons, cytokines and retinoids [23,24,25,26]. In addition, *IRF-1* has been recognized to play a role in innate and adaptive immunity [23,24]. Previous studies suggest that *IRF-1* has an anti-tumor effect through growth inhibition and induction of apoptotic genes including caspase, tumor necrosis factor-related apoptosis-inducing ligand (TRAIL), and lysyl oxidase (LOX) [27]. In human articular chondrocytes specifically, *IRF-1* has been reported to mediate IL1β-induced stimulation of *MMP3* and *MMP13* [28] and TNFα-induced collagen 2 degradation and *MMP13* stimulation [29]. However, the role of *IRF-1* of chondrocytes on cell death remains unclear. The IPA canonical pathway analysis revealed an interesting interaction between retinoid and interferon (IFN) signaling pathways. Our findings revealed a number of downstream molecules of interferon receptors and STAT2, a mediator of *IFN* signaling, were up-regulated in the RARγ-treated explant cultures. Since the up-regulation of these downstream genes was also found in polydactyly chondrocytes, the responding cells are most likely osteochondroma chondrocytes, but not other contaminated cells such as bone marrow cells. The cross-talk between these two pathways has been demonstrated in other cells types. In renal cancer cells, 13-cis-retinoic acid and IFN-alpha plus IFN-gamma showed additive inhibitory actions on cell growth [30]. In myeloid cell line cells, retinoic acids stimulate expression of STAT1, STAT2 and p48, which are involved in IFN-alpha signaling [31]. The significance of this cross-talk in chondrocytes and osteochondroma cells should be further studied.

In summary, this study demonstrated that RARγ agonists target human osteochondroma cells and induce various biological responses including proteoglycan degradation and cell death induction. Although these results are promising, it would be too early to reach definitive conclusions regarding the pharmacological effect of RARγ agonists on human osteochondromas, since the number of tested samples was limited. In addition, we also need to be cautious about potential side-effects of RARγ agonists on growing skeletal tissues as suggested previously [32]. However, the findings support the hypothesis that RARγ agonists exert anti-tumor function on osteochondromas and encourages current and future studies on the development of pharmacological therapy for osteochondromas.

## 4. Materials and Methods

### 4.1. Human Osteochondroma Specimens

Osteochondroma specimens were obtained during surgical procedures through the collaboration with of the Pathology Biorepository Shared Service (PBSS) of osteochondromas (seven patients, five multiple osteochondromas and two sporadic osteochondromas) (Table S1) under the IRB protocols (University of Maryland, Baltimore and Sinai Hospital of Baltimore). The specimens were micro-dissected into 3–5 mm cubic pieces and cultured onto the cell culture insert (Corning, Tewksbury, MA, USA) in high-glucose Dulbecco’s Modified Eagle Medium (DMEM) (Corning, Manassas, VA, USA) containing 2% charcoal-treated FBS. The explants were treated with 50 or 300 nM NRX 204647 (RARγ agonist), 300 nM Palovarotene or vehicle for four or seven days.

### 4.2. Chondrocytes from Human polydactyly 

Polydactyly specimens were also obtained at different surgical procedures and chondrocytes were isolated. These specimens were determined to be non-human subjects due to the typical discarding of the specimens. After dissection of the cartilaginous portion of the specimens, chondrocytes were dissociated by incubation of 0.5 mg/mL type I collagenase in serum-free DMEM overnight at 37 °C. The cells were plated at 7.8 × 10^4^ cells /cm^2^, cultured in 10% DMEM until confluency and treated with 300 nM NRX 204647 (RARγ agonist) in high-glucose DMEM containing 2% charcoal-treated FBS.

### 4.3. Drug and Drug Treatment

NRX204647 (CAS131452-80-6) [33] and Palovarotene (CAS410528-02-8) [14] were synthesized at Atomax Chemicals Company Limited (Shenzhen, China). Drug quality and activity were confirmed by mass spectrometry and the reporter assay using the RARγ Reporter Cellular Assay Pack (BPS Bioscience, San Diego, CA, USA). NRX-204647 and Palovarotene share a common backbone and activate retinoic acid nuclear (RARγ) gamma receptor with higher specificity than that to RARα or RARβ, and the biological effective dose of NRX-204647 is lower than that of Palovarotene [14]. The explant or cell cultures were treated with 50–300 nM NRX204647 or 300 nM Palovarotene every other day 0.1% ethanol was added to the control cultures.

### 4.4. Histology

Osteochondroma explants were fixed 4% PFA overnight at 4 °C and embedded in paraffin. Serial sections (5 μm) were made and subjected to Hematoxylin and Eosin staining and alcian blue staining (pH 1.0) (*n* = 3–4/patient, four patients). The sections were also subjected to immunostaining to detect the presence of the neo-epitope of aggrecan (NITEGE and VDIPEN, provided by Dr. J. Mort, Shriners Hospital, Montreal, Quebec, Canada), SOX9 (AB5535, 1:200, EMD Millipore, Burlington, MA, USA). For NITEGE (1:1000) and VDIPEN (1:400) staining, the sections were incubated with 1% hyaluronidase for 30 min at 37 °C. The primary antibodies on the sections were visualized by incubation with peroxidase-conjugated proper secondary antibodies (Vector Laboratories, Burlingame, CA, USA) followed by DAB (3,3′-Diaminobenzidine) staining using ImmPactDAB Peroxidase Substrate kit (Vector Laboratories). The sections were counterstained with methyl blue. The images were observed under the microscope (BXZ-700, Keyence, Itasca, IL, USA), captured and analyzed by Cell Hybrid Count function in BZX-700. Two to three sections per individual sample were used to analyze the staining results. Statistical analysis was performed using unpaired t-test or one-way analysis of variance (ANOVA) and Tukey’s multiple comparison tests using Prism 6 (GraphPad Software, La Jolla, CA, USA).

### 4.5. Live/Dead Assay

Osteochondroma explants (*n* = 3/patients, 3 patients) were washed with PBS twice and incubated with 2 μM green-fluorescent calcein-AM (Thermo Fisher Scientific, Waltham, MA, USA) and 1 μM red-fluorescent ethidium homodimer-1 (Thermo Fisher Scientific, Waltham, MA, USA) for 5 min at room temperature. The images were observed under the microscope (BXZ-700) and captured.

### 4.6. RNA isolation and qPCR

Osteochondroma explants were treated with vehicle, 50 or 300 nM NRX204647 or 300 nM Palovarotene for 4 days. They were washed with PBS twice, soaked in RNAlater stabilization solution (Thermo Fisher Scientific, Waltham, MA, USA) and stored at −80 °C until RNA preparation. To isolate total RNA, tissues were placed on clean Kimwipe to absorb excess RNAlater solution and then were snap-frozen in liquid nitrogen. Frozen tissue fragments were placed on pre-chilled carbon steel block and crashed into powder by the steel hammer. We then purified total RNA using Qiagen RNeasy fibrous tissue mini kit (Qiagen, Germantown, MD, USA). 

Purified total RNA was reverse transcribed (SuperScript IV VILO, ThermoFisher Sciences) and subjected to qPCR using the validated PrimeTime qPCR primers (Integrated DNA Technology, Coralville, IA): *ACTB*, Hs.PT.39a.22214847; *ACAN*, Hs.PT.56a.742783; *COL2A1*, Hs.PT.58.4107778; *COL9A1*, Hs.PT.58.38805503; *SOX9*, Hs.PT.58.38984663; *MMP13*, Hs.PT.58.4073501; *TGM2*, Hs.PT.58.3966447; *CYP26B1*, Hs.PT.58.38517191. qPCR was performed with an Applied Biosystems 7900HT Sequence Detection Systems running SDS 2.1 software using SYBR green reagents (Applied Biosystems, Foster City, CA). The average threshold cycle value (Ct value) was calculated from quadruplicate reactions. Standard curves were generated using 10-fold serial dilutions of cDNA of each gene with a correlation coefficient of >0.98. Relative expression levels were calculated based on a standard curve and normalized to beta-Actin (*ACTB*).

### 4.7. Transcriptome Analysis

Library preparation, sequencing and data analysis were performed by the Genomic Resource Center (University of Maryland Baltimore), the Genewiz (South Plainfield, NJ, USA) or the Genome Sciences (Penn State University College of Medicine). We analyzed all sets of osteochondroma together and an individual specimen of RNAseq data. In the former case, BBDuk (https://jgi.doe.gov/data-and-tools/bbtools/bb-tools-user-guide/bbduk-guide/) was used to trim/filter Illumina adapters and low-quality sequences using “qtrim=lr trimq=10 maq=10” option. Next, the alignment of the filtered reads to the human reference genome (GRCh38) was done using HISAT2 v2.1.0 (https://ccb.jhu.edu/software/hisat2/manual.shtml) [34] applying –no-mixed and –no-discordant options. Read counts were calculated using HTSeq [35] by supplementing Ensembl gene annotation (GRCh38.78). Gene expression values were calculated as counts per million (CPM). Genes with CPM > 0.1 in at least three samples were included. EdgeR (https://bioconductor.org/packages/release/bioc/html/edgeR.html) [36] was used to fit the read counts to the negative binomial model along with the generalized linear model (GLM). The design matrix was built to account for the batch effect and differentially expressed genes were determined by the likelihood ratio test method by adjusting for differences between batches. Significance was defined to be those with q-value < 0.05 calculated by the Benjamini-Hochberg method to control the FDR. To analyze an individual set of RNA sequence data (OC5, 6, 7, 9 and 10, and polydactyly sample), sequence reads were trimmed to remove possible adapter sequences and nucleotides with poor quality using Trimmomatic v.0.36. The trimmed reads were mapped to the Homo sapiens GRCh38 reference genome available on ENSEMBL using the STAR aligner v.2.5.2b (https://github.com/alexdobin/STAR). Using DESeq2 (https://bioconductor.org/packages/release /bioc/html/DESeq2.html), a comparison of gene expression between the customer-defined groups of samples was performed. The Wald test was used to generate *p*-values and log2 fold changes. Genes with an adjusted *p*-value < 0.05 and absolute log2 fold change > 1 were called as differentially expressed genes for each comparison. Pathway analysis was performed with Ingenuity pathway analysis software (Qiagen) and DAVID Bioinformatics Resources 6.8. 

## Figures and Tables

**Table 1:** Analysis of gene ontology (GO) biological process of differentially expressed genes (DEGs).

GO ID	Description	*p*-Value	Gene
0030198	extracellular matrix organization	2.60 × 10^−24^	*GFAP, TNC, NPNT, ELN, ITGA11, COL2A1, POSTN, ITGB3, SOX9, ABI3BP, COL9A1, SMOC2, TNFRSF11B, COL9A2, COL9A3, COL7A1, COL27A1, COMP, COL6A3, ACAN, COL6A1, LOX, FGF2, COL8A2, COL11A1, LOXL1, CYR61, COL10A1, ICAM1, HAPLN1, MATN3, MATN4, MATN1, ITGA1, HSPG2, ITGA4, SPARC, COL5A3, COL16A1, NDNF, COL5A1, CSGALNACT1, COL14A1, BGN, COL19A1, ITGA6, LAMC3, COL1A2, COL1A1*
0001501	skeletal system development	6.50 × 10^−16^	*ALPL, NOG, FGFR3, PTH1R, SOX4, POSTN, COL2A1, EXTL1, SOX9, COL9A2, TNFRSF11B, LECT1, COMP, ACAN, COL12A1, COL11A2, PAPSS2, COL10A1, MATN3, HAPLN1, BMP2, CMKLR1, IGF2, ANKH, EPHA2, PRELP, SHOX2, COL19A1, CLEC3A, ETS2, COL1A2, FOXC1, COL1A1*
0030199	collagen fibril organization	8.49 × 10^−13^	*FMOD, ADAMTS14, COL2A1, COL5A3, GREM1, SERPINH1, COL5A1, COL14A1, SFRP2, ACAN, COL1A2, COL12A1, FOXC1, COL1A1, LOX, COL11A2, COL11A1*
0007155	cell adhesion	3.01 × 10^−11^	*NRP2, MYBPC1, IGFBP7, POSTN, CTNNB1, WISP2, CGREF1, S1PR1, SRPX, WISP3, COL12A1, BOC, CYR61, ICAM1, TYRO3, ACKR3, CTNNA3, HES1, AMBP, LAMC3, CX3CR1, COL1A1, MFAP4, TNC, ITGA11, CTNND2, ITGB3, CX3CL1, ISLR, LPXN, COL7A1, COMP, COL6A3, ACAN, PSTPIP1, COL6A1, CD24, THBS2, THBS3, HAPLN1, FLRT1, COL15A1, ITGA4, COL16A1, TINAGL1, COL5A1, CDH13, COL19A1, CDH15, ITGA6, DSG2, ENG, MYH10*
0030574	collagen catabolic process	4.94 × 10^−10^	*ADAMTS14, COL15A1, COL2A1, MMP16, COL5A3, MMP13, COL5A1, COL19A1, COL7A1, COL6A3, COL1A2, COL12A1, COL6A1, COL1A1, COL11A2, COL8A2, COL11A1, COL10A1*
0060337	type I interferon signaling pathway	4.22 × 10^−9^	*BST2, IFITM1, IFITM3, OAS3, OAS1, OAS2, IFI35, STAT2, IFIT3, IFIT2, OASL, ISG15, IRF1, IRF2, MX1, GBP2, IFI6*

**Table 2:** Canonical pathways of DEGs of osteochondromas by the ingenuity pathway analysis.

Pathways	−log (*p*-Value)	Ratio	Gene
Hepatic Fibrosis/Hepatic Stellate Cell Activation	14.9	0.183	*A2M, ACTA2, COL10A1, COL11A1, COL11A2, COL12A1, COL15A1, COL16A1, COL19A1, COL1A1, COL1A2, COL21A1, COL27A1, COL2A1, * *COL5A1, COL5A3, COL6A1, COL6A3, COL7A1, COL8A2, COL9A1, COL9A2, COL9A3, CSF1, FGF2, ICAM1, IGF1R, IGF2, IL10, LBP, MMP13, M H10, TIMP1, TNFRSF11B*
GP6 Signaling Pathway	13.4	0.218	*COL10A1, COL11A1, COL11A2, COL12A1, COL15A1, COL16A1, COL19A1, COL1A1, COL1A2, COL21A1, COL27A1, COL2A1, COL5A1, COL5A3, COL6A1, COL6A3, COL7A1, COL8A2, COL9A1, COL9A2, COL9A3, ITGB3, LAMC3, PRKCH, PRKCZ, SCHIP1*
Atherosclerosis Signaling	7.91	0.159	*ALOX15, APOL1, COL10A1, COL11A2, COL1A1, COL1A2, COL2A1, COL5A3, CSF1, ICAM1, ITGA4, MMP13, PLA2G12A, PLA2G3, PLA2G5, PLAAT3, PLAAT4, PNPLA3, RBP4, SERPINA1*
Osteoarthritis Pathway	7.07	0.118	*ACAN, ADAMTS5, ALPL, ANKH, BMP2, CASP4, CNMD, COL10A1, COL2A1, CTNNB1, FGF2, FGFR3, FZD5, FZD9, GLI3, GREM1, H19, HES1, ITGA4, MATN3, MMP13, NOS2, PTH1R, RBP4, SOX9*
Interferon Signaling	6.59	0.278	*IFI35, IFI6, IFIT3, IFITM1, IFITM3, IRF1, ISG15, MX1, OAS1, STAT2*

**Table 3:** Canonical pathways of DEGs of polydactyly chondrocytes by the ingenuity pathway analysis.

Pathways	−log (*p*-Value)	Ratio	Molecules
Hepatic Fibrosis/Hepatic Stellate Cell Activation	10.7	0.231	*A2M, AGTR1, CCL2, CCN2, CD14, COL11A1, COL16A1, COL17A1, COL1A1, COL21A1, COL23A1, COL27A1, COL2A1, COL4A3, COL4A5, COL4A6, COL5A1, COL5A3, COL6A1, COL6A6, COL9A1, COL9A2, COL9A3, CSF1, CXCL8, HGF, IGF1, IGF2, IL1B, IL1R2, IL1RL1, IL1RL2, LBP, LY96, MET, MMP1, MMP13, MYH14, TGFB2, TIMP1, TLR4, TNFRSF11B, VEGFD*
Atherosclerosis Signaling	8.18	0.242	*ALOX12B, ALOX15B, ALOXE3, APOD, APOL1, CCL2, CLU, COL1A1, COL2A1, COL5A3, CSF1, CXCL8, IL18, IL1B, IL36G, IL36RN, ITGA4, LPL, MMP1, * *MMP13, PLA2G2A, PLA2G3, PLA2G4D, PLA2G4E, PLA2G4F, PLAAT3, PLAAT4, RBP4, S100A8, SELP*
Osteoarthritis Pathway	0.774	0.192	*ACAN, ADAMTS5, ALPL, CASP14, CASP4, CNMD, COL2A1, CTNNB1, CXCL8, DKK1, ELF3, FGF18, FGFR3, FRZB, FZD5, FZD9, GDF5, H19, HES1, IHH, IL1B, IL1R2, IL1RL1, IL1RL, ITGA4, MATN3, mir-140, MMP1, MMP13, NOS2, PPARGC1A, PRKAG2, PTHLH, RARRES2, RBP4, S100A8, S100A9, SERPINA12, SOX9, TLR4, VEGFD*
LPS/IL-1 Mediated Inhibition of RXR Function	0.710	0.183	*ABCB1, ABCG1, ACSL5, ALDH1A1, ALDH1L1, ALDH1L2, ALDH3A1, ALDH3B2, CD14, CHST3, CPT1A, CYP3A5, FABP5, FABP7, FMO1, FMO2, FMO4, GSTO2, HS3ST1, HS3ST3A1, HS6ST2, IL18, IL1B, IL1R2, IL1RL1, IL1RL2, IL36G, IL36RN, IL4I1, LBP, LY96, MAOB, NR1H3, PAPSS2, PPARGC1, SCARB1, SLC27A2, SMOX, SULT2B1, TLR4, TNFRSF11B*
Role of Osteoblasts, Osteoclasts and Chondrocytes in Rheumatoid Arthritis	0.681	0.181	*ADAMTS5, ALPL, APC2, BMP10, BMP3, BMP4, BMP6, BMP7, CALML5, CAMK4, COL1A1, CSF1, CTNNB1, DKK1, FOS, FRZB, FZD5, FZD9, IGF1, IL18, IL1B, IL1R2, IL1RL1, IL1RL2, IL36G, IL36RN, ITGB3, MAP2K6, MAP3K5, MMP1, MMP13, SFRP2, SFRP4, SFRP5, TNFRSF11B, TNFSF11, WNT10A, WNT2B, WNT4, WNT7A*

## Supplementary Materials

Supplementary materials can be found at https://www.mdpi.com/1422-0067/21/8/2686/s1.
