# Supplementary material for: Understanding the Action of RARγ Agonists on Human Osteochondroma Explants
Source: Int J Mol Sci. 2020 Apr 13;21(8):2686. doi: 10.3390/ijms21082686 (PMC7215996; doi:10.3390/ijms21082686)
Supplement: Supplementary file 1 [file ijms-21-02686-s001.zip › Supplementary data/Updated_SG_IJMS_SupplementaryFigures/FINAL_Supplement materials.pdf]

A. hOC5

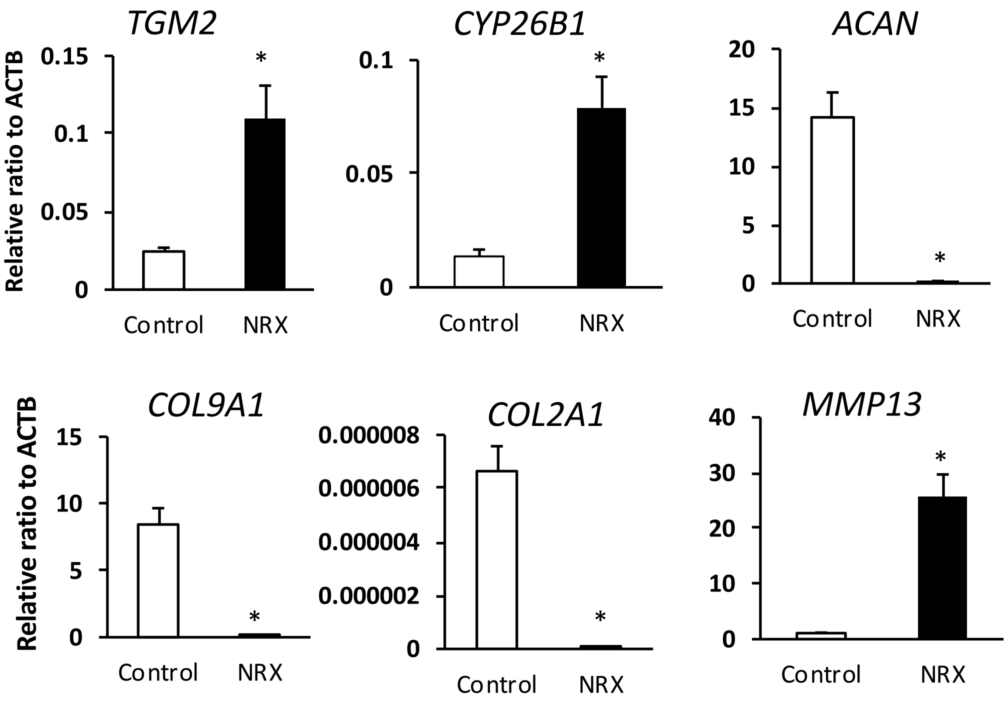

B. hOC6

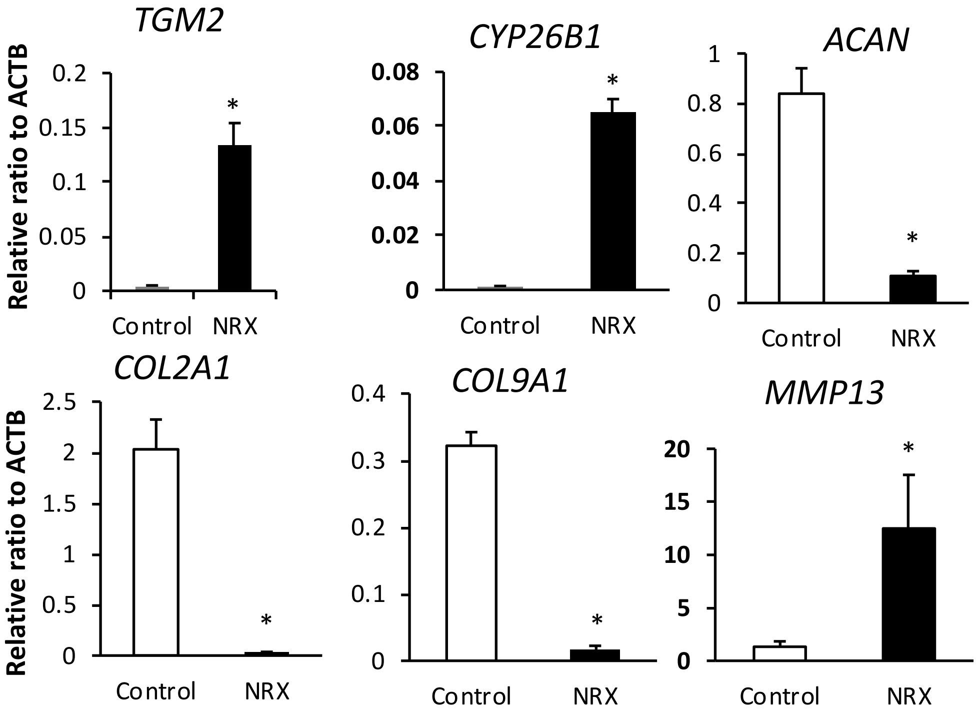

Supplement Figure 1. Gene expression analysis of RARγ agonist treatment and control groups of osteochondroma explant cultures (OC5 and OC6). The osteochondroma explants (OC5 and 6) were treated with vehicle (0.1% ethanol) and 300 nM NRX204647 in DMEM containing 2% charcoal-treated FBS (n=3). Total RNAs were prepared and subjected to qPCR to examine gene expression levels of *CY26B1, TGM2, ACAN, COL2A1, COL9A1* and *MMP13.* The values are average and standard deviation (SD). * $p < 0.05$ versus control.

Supplement table 5. Top 5 canonical pathways ranked by IPA

hOC5

Pathways	-log(p-value)	Ratio	Molecules
Osteoarthritis Pathway	20.0	0.322	*ACAN,ACVRL1,ADAMTS5,ALPL,ANKH,ANXA2,BGLAP,BMP2,CASP4,CNMD,COL10A1,COL2A1,CREB3,CREBBP,CTNNB1,DDIT4,DDR2,DLX5,FGF2,FGFR1,FGFR3,FOXO3,FRZB,FZD5,FZD7,FZD9,GLI2,GLI3,GLIS2,GREM1,H19,HDAC4,HES1,IHH,IL1R1,IL1RAP,ITGA2,ITGA4,JAG1,LEF1,MATN3,MEF2C,MMP13,MMP3,MMP9,NAMPT,PDGFC,PGF,PPARG,PRKAB2,PTCH1,PTH1R,RBP4,RUNX2,SIRT1,SLC39A8,SMAD1,SMAD6,SMAD9,SOX9,SPHK1,SPP1,TCF4,TCF7,TIMP3,TNFRSF1A,TNFRSF1B,VEGFA*
EIF2 Signaling	14.2	0.272	*ACTA2,ACTB,AGO1,ATF3,ATF5,CDK11A,DDIT3,EIF1,EIF2B5,EIF2S1,EIF2S2,HRAS,HSPA5,MRAS,MYC,PIK3CA,PIK3CG,PIK3R5,PPP1R15A,RALA,RASD1,RPL10,RPL10A,RPL12,RPL15,RPL17,RPL18A,RPL21,RPL22L1,RPL26,RPL29,RPL30,RPL31,RPL32,RPL34,RPL35,RPL35A,RPL36,RPL37A,RPL39,RPL41,RPL5,RPL7,RPL8,RPS12,RPS13,RPS15A,RPS17,RPS18,RPS23,RPS27A,RPS29,RPS3,RPS5,RPS6,RPS8,RPSA,SHC1,TRIB3,VEGFA,WARS1*
Hepatic Fibrosis Signaling Pathway	9.46	0.198	*ACTA2,CCN2,CDC42,COL10A1,COL11A2,COL18A1,COL1A1,COL2A1,CREB3,CREBBP,CTNNB1,DVL2,EDNRA,EZH2,FGF2,FGFR1,FOXO1,FZD5,FZD7,FZD9,GLI2,GLI3,GLIS2,HRAS,ICAM1,IKBKE,IL1R1,IL1RAP,ITGA2,ITGA4,ITGB3,JAK2,LEF1,MAP2K5,MAP2K6,MMP13,MRAS,MYC,MYD88,MYLK,PDCD4,PDGFA,PDGFC,PDGFD,PDGFRB,PGF,PIK3CA,PIK3CG,PIK3R5,PPARG,PRKCZ,PTCH1,RALA,RASD1,RHOBTB2,RHOU,RND3,SOD2,SPP1,STAT3,TCF4,TCF7,TGFB2,TGFB3,TIMP1,TNFRSF11B,TNFRSF1A,TNFRSF1B,VCAM1,VEGFA,WNT11,WNT3,WNT5A*
Human Embryonic Stem Cell Pluripotency	8.96	0.274	*BDNF,BMP2,BMP5,BMP6,CTNNB1,FGF2,FGFR1,FGFR2,FGFR3,FGFR4,FGFRL1,FOXO1,FZD5,FZD7,FZD9,LEF1,MRAS,NOG,PDGFA,PDGFC,PDGFD,PDGFRB,PIK3CA,PIK3CG,PIK3R5,S1PR1,SALL4,SMAD1,SMAD6,SPHK1,TCF4,TCF7,TGFB2,TGFB3,WNT11,WNT3,WNT5A*
Regulation of the Epithelial-Mesenchymal Transition Pathway	8.35	0.234	*CDH2,CTNNB1,DVL2,EGR1,ETS1,FGF2,FGFR1,FGFR2,FGFR3,FGFR4,FGFRL1,FOXC2,FZD5,FZD7,FZD9,HRAS,ID2,JAG1,JAK2,JAK3,LEF1,MAP2K5,MAP2K6,MMP9,MRAS,NOTCH2,NOTCH3,PDGFD,PDGFRB,PIK3CA,PIK3CG,PIK3R5,RALA,RASD1,SMURF1,SNAI2,STAT3,TCF4,TCF7,TGFB2,TGFB3,WNT11,WNT3,WNT5A,ZEB2*

hOC6

Pathways	-log(p-value)	Ratio	Molecules
Hepatic Fibrosis / Hepatic Stellate Cell Activation	10.2	0.237	*A2M,ACTA2,AGTR1,CCL2,CD40,COL10A1,COL11A1,COL11A2,COL12A1,COL15A1,COL16A1,COL1A1,COL1A2,COL2A1,COL4A1,COL5A1,COL6A3,COL7A1,COL8A2,COL9A1,COL9A2,COL9A3,CSF1,FGF2,FN1,ICAM1,IFNAR2,IFNGR2,IGF1,IGF1R,IGF2,IL10,IL4R,LBP,MMP13,MYH10,NFKB2,PGF,SMAD4,TIMP1,TLR4,TNFRSF11B,TNFRSF1B,VCAM1*
Interferon Signaling	9.33	0.472	*IFI6,IFIT1,IFIT3,IFITM1,IFITM3,IFNAR2,IFNGR2,IRF1,ISG15,JAK1,JAK2,MX1,OAS1,PSMB8,PTPN2,STAT2,TAP1*
Osteoarthritis Pathway	8.88	0.213	*ACAN,ADAMTS5,ANKH,BMP2,BMPR1A,CASP4,CEBPB,CNMD,COL10A1,COL2A1,CTNNA1,DDIT4,FGF2,FGFR3,FN1,FRZB,FZD1,FZD4,FZD5,FZD6,FZD7,GLI3,GREM1,H19,HDAC4,HES1,ITGA4,ITGA5,JAG1,MATN3,MEF2C,MMP13,MYBBP1A,NFKB2,PGF,PRKAA2,PTGS2,RARRES2,SMAD4,SMAD9,SOX9,TCF7L1,TLR4,TNFRSF1B,ZNF875*
Hepatic Fibrosis Signaling Pathway	5.85	0.155	*ACTA2,ACVR2B,AGTR1,CACNA1C,CALM1 (includes others),CCL2,CD40,CEBPB,COL10A1,COL11A2,COL1A1,COL1A2,COL2A1,DIRAS3,ELK1,FGF2,FOS,FZD1,FZD4,FZD5,FZD6,FZD7,GLI3,ICAM1,IKBKB,IL17RA,ITGA4,ITGA5,ITGB3,JAK1,JAK2,MAP2K1,MAP2K3,MMP13,MRAS,NFKB2,NFKBID,NOX4,OPN1SW,PGF,PRKD1,RASD1,RHOB,RHOBTB1,SDHB,SMAD4,SOS2,TCF7L1,TF,TIMP1,TIRAP,TLR4,TNFRSF11B,TNFRSF1B,VCAM1,WNT5A,ZNF875*
GP6 Signaling Pathway	5.63	0.218	*CALM1,COL10A1,COL11A1,COL11A2,COL12A1,COL15A1,COL16A1,COL1A1,COL1A2,COL2A1,COL4A1,COL5A1,COL6A3,COL7A1,COL8A2,COL9A1,COL9A2,COL9A3,GRAP2,ITGB3,ITPR1,LAMA3,LAMB3,LAMC2,LYN,PRKD1*

hOC7

Pathways	-log(p-value)	Ratio	Molecules
T Helper Cell Differentiation	6.99	0.219	*CD40,CD86,GATA3,HLA-DMA,HLA-DMB,HLA-DQA1,HLA-DQB1,HLA-DRA,HLA-DRB1,HLA-DRB5,IFNGR1,IL10RA,IL21R,STAT1,STAT6,TNFRSF11B*
Th1 Pathway	6.41	0.165	*CD4,CD40,CD86,DLL1,GATA3,HAVCR2,HLA-DMA,HLA-DMB,HLA-DPA1,HLA-DPB1,HLA-DQA1,HLA-DQB1,HLA-DRA,HLA-DRB1,HLA-DRB5,IFNAR1,IFNGR1,IL10RA,NFATC1,STAT1*
Th1 and Th2 Activation Pathway	6.20	0.140	*BHLHE41,CD4,CD40,CD86,DLL1,GATA3,HAVCR2,HLA-DMA,HLA-DMB,HLA-DPA1,HLA-DPB1,HLA-DQA1,HLA-DQB1,HLA-DRA,HLA-DRB1,HLA-DRB5,IFNAR1,IFNGR1,IKZF1,IL10RA,NFATC1,STAT1,STAT6,TGFBR3*
Antigen Presentation Pathway	6.16	0.282	*CD74,CIITA,HLA-DMA,HLA-DMB,HLA-DPA1,HLA-DPB1,HLA-DQA1,HLA-DQB1,HLA-DRA,HLA-DRB1,HLA-DRB5*
B Cell Development	5.59	0.278	*CD40,CD86,HLA-DMA,HLA-DMB,HLA-DQA1,HLA-DQB1,HLA-DRA,HLA-DRB1,HLA-DRB5,IL7R*

hOC9

Pathways	-log(p-value)	Ratio	Molecules
Hepatic Fibrosis / Hepatic Stellate Cell Activation	9.22	0.140	*ACTA2,AGTR1,CD14,COL16A1,COL1A1,COL2A1,COL4A1,COL4A2,COL5A1,COL5A3,COL6A1,COL6A2,COL6A3,COL7A1,COL8A2,COL9A1,FGF1,FGFR1,ICAM1,IGFBP5,IL10,IL10RA,MMP9,MYH10,TIMP1,TNFRSF1B*
GP6 Signaling Pathway	7.11	0.151	*COL16A1,COL1A1,COL2A1,COL4A1,COL4A2,COL5A1,COL5A3,COL6A1,COL6A2,COL6A3,COL7A1,COL8A2,COL9A1,FCER1G,LAMA3,PIK3R5,PRKCB,SYK*
Hepatic Fibrosis Signaling Pathway	5.65	0.084	*ACTA2,AGTR1,COL1A1,COL2A1,COL5A3,CTNNB1,CYBB,FGFR1,FOS,FOXO4,FZD10,FZD3,FZD4,GLIS1,ICAM1,IL1RN,IL33,IRAK3,ITGA4,KLF9,PIK3R5,PRKACA,PRKAG2,PRKAR2B,PRKCB,TCF7L1,TF,TIMP1,TNFRSF1B,WNT5B,WNT7B*
Osteoarthritis Pathway	5.65	0.104	*ACAN,ADAMTS5,ALPL,CNMD,COL2A1,CTNNB1,FGFR1,FZD10,FZD3,FZD4,GLIS1,H19,ITGA4,JAG1,MATN3,MMP9,P2RX7,PRKAA2,PRKAG2,TCF7L1,TIMP3,TNFRSF1B*
Cell Cycle: G2/M DNA Damage Checkpoint Regulation	5.41	0.204	*AURKA,BRCA1,CCNB1,CCNB2,CDK1,CKS2,GADD45A,PLK1,RPS6KA1,TOP2A*

hOC10

Pathways	-log(p-value)	Ratio	Molecules
Hepatic Fibrosis / Hepatic Stellate Cell Activation	14.4	0.215	*ACTA2,COL10A1,COL11A1,COL12A1,COL15A1,COL16A1,COL1A1,COL1A2,COL21A1,COL27A1,COL2A1,COL3A1,COL4A1,COL4A2,COL4A5,COL5A1,COL5A2,COL5A3,COL6A1,COL6A3,COL7A1,COL8A2,COL9A1,COL9A2,COL9A3,CSF1,ICAM1,IGF2,IGFBP5,IL10,IL1RL1,MMP1,MYH14,PDGFD,PDGFRB,SERPINE1,TGFB2,TGFB3,TGFBR2,TIMP1*
GP6 Signaling Pathway	13.6	0.258	*COL10A1,COL11A1,COL12A1,COL15A1,COL16A1,COL1A1,COL1A2,COL21A1,COL27A1,COL2A1,COL3A1,COL4A1,COL4A2,COL4A5,COL5A1,COL5A2,COL5A3,COL6A1,COL6A3,COL7A1,COL8A2,COL9A1,COL9A2,COL9A3,FGG,ITGB3,LAMA3,LAMB2,LCP2,PIK3CD,PRKCZ*
Osteoarthritis Pathway	7.52	0.150	*ACAN,ADAMTS5,ALPL,ANKH,CASP12,CASP4,CNMD,COL10A1,COL2A1,DDR2,FGFR3,FZD5,FZD7,H19,HES1,HIF1A,IHH,IL1RL1,ITGA4,MATN3,MMP1,NKX3-2,NOS2,PRKAB2,PTH1R,RBP4,SMAD1,SOX9,SP7,SPHK1,SPP1,TGFBR2*
Interferon Signaling	6.99	0.333	*IFI35,IFI6,IFIT1,IFIT3,IFITM1,IFITM2,IFITM3,IRF1,ISG15,MX1,OAS1,STAT2*
Atherosclerosis Signaling	6.02	0.169	*APOD,APOL1,COL10A1,COL1A1,COL1A2,COL2A1,COL3A1,COL5A3,CSF1,ICAM1,IL33,ITGA4,LPL,MMP1,PDGFD,PLA2G12A,PLA2G2A,PLAAT3,PLAAT4,RBP4,SERPINA1*
